# Synthesis and Thermotropic Studies of a New Series of Teraryl Liquid Crystals 2-(4′-Alkoxybiphen-4-yl)-5-Cyanopyridines

**DOI:** 10.3390/ijms140918809

**Published:** 2013-09-12

**Authors:** Win-Long Chia, Xue-Ming Lin

**Affiliations:** Department of Chemistry, Fu Jen Catholic University, New Taipei City 24205, Taiwan; E-Mail: sam205131@yahoo.com.tw

**Keywords:** liquid crystals, teraryl compounds, cyanopyridine terminus, mesomorphic behavior

## Abstract

A new series of teraryl 2-(4′-alkoxybiphen-4-yl)-5-cyanopyridines (*nO-*PPPyCN, *n* = 2–8) compounds, bearing a cyanopyridine terminus, were synthesized using a short 2-step reaction with overall yields between 33% and 69%. Spectral analyses were in accord with the expected structures. Thermotropic behavior of the teraryl compounds was investigated using polarised optical microscopy and differential scanning calorimetry. All compounds exhibited both enantiotropic nematic and smectic A phases, with an additional enantiotropic smectic C phase when *n* = 7 and 8. Polymesomorphism appears to be a common behavior in this series of linear liquid crystal compounds.

## 1. Introduction

Since the discovery of liquid crystals (LC), it has become apparent that molecular structure is crucial to the liquid crystalline state. During the early twentieth century, Daniel Volander, a synthetic chemist, proposed that the crystalline-liquid (mesomorphic) state occurs when a molecular structure is as linear as possible [1]. Thus, liquid-crystal molecular structures are typically designed and constructed based on the crude principle that the shape anisotropy of a mesogen is rod-like. Additionally, synthesis provides a useful tool to investigate the factors that influence the formation of liquid crystals.

Advancements in thermal analysis instrumentation, such as controllable and programmable heating and cooling rates, and improvements in enthalpy measurement accuracy, have allowed scientists to correlate the structures of liquid-crystal molecules with their physical properties. Thus, it is now possible to investigate the subtle and sophisticated influences of the various moieties within a liquid crystalline molecule.

Because of difficulties with synthesis, relatively few studies have researched the thermotropic trends within homologous series of pyridine-containing liquid crystalline compounds [2–5]. The synthesis of pyridine-containing LCs typically involves a cyclization reaction between an enamine and a vinyl ketone to furnish a dihydropyran derivative, which then reacts with hydroxylamine hydrochloride to form a pyridine [3]. Another route to 2,5-disubstituted pyridines is the condensation of an acetophenone with ethyl formate in the presence of sodium, followed by cyclization with cyanoacetamide, substitution of oxygen by chlorine, and then reductive elimination of the chlorine [6–8].

Pyridine-containing liquid crystalline compounds have also been prepared using several other methods [9–14], for example, by cross coupling of arylboronic acids with halopyridines in the presence of a palladium complex [9–11], or by the reaction of 2,2-dichloro-1-(4-methylphenyl)-cyclopropane carbaldehyde and a 4-*n*-alkoxybenzyl amine at an elevated temperature [12–14]. Recent advances in organometallic coupling-based methods, using trifluoroborate derivatives, such as those reported by Molander *et al.* [15,16], have been particularly successful in the coupling of heterocyclic moieties. Seed *et al.* [17] used a modification of this method to prepare a new class of mesogenic materials that exhibit the smectic C phase. Although such methods are valuable in preparing critical heterocyclic LC molecules, most suffer from a limited scope, the need for a relatively expensive catalyst, and occasionally a large number of low-yield synthetic steps. Thus, an inexpensive and simple synthetic method is required to fully investigate the influence of heteroaryl structural moieties on the physical properties of liquid crystalline materials.

We previously reported a novel method for the synthesis of pyridine-containing liquid crystalline compounds [18–21]. Here, we report a convenient, short 2-step method for the synthesis of a homologous series of 2-(4′-alkoxybiphen-4-yl)-5-cyanopyridines (*nO-*PPPyCN), in which *n* varies from 2 to 8 (ethoxy to octoxy). Additionally, we correlate the length of the alkoxy chain with thermotropic behavior for this homologous series. The molecules designed in this study contain an alkoxy tail and a teraryl mesogen, consisting of a biphenyl moiety and a cyanopyridyl terminus.

## 2. Results and Discussion

### 2.1. Synthesis

**Scheme 1:**
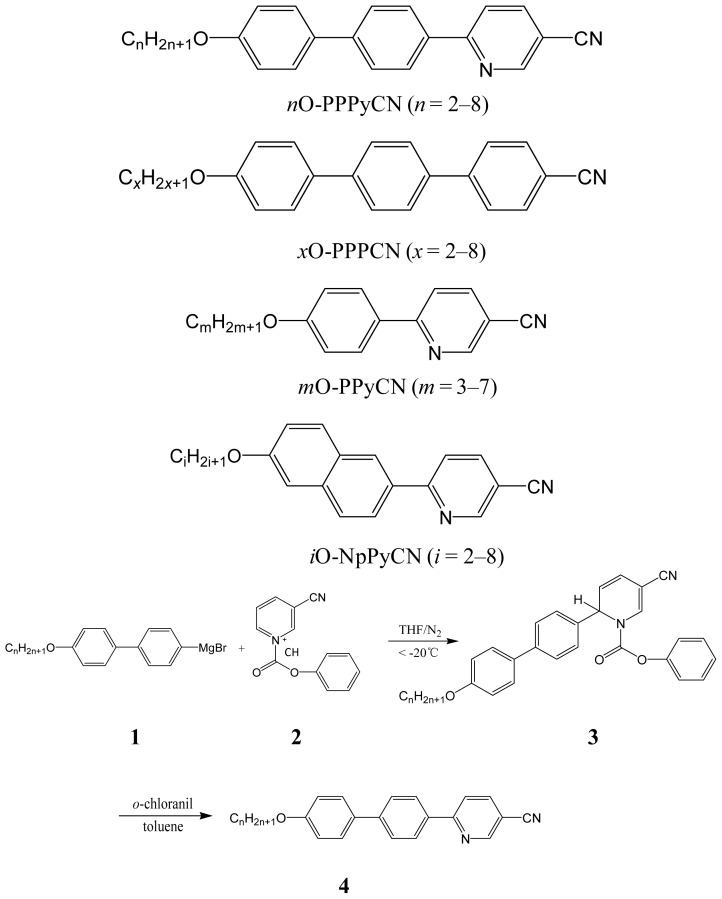
Synthetic route for the synthesis of *nO-*PPPyCN.

A series of 2-(4′-alkoxybiphen-4-yl)-5-cyanopyridines (*nO-*PPPyCN, *n* = 2–8) were obtained first by the regioselective addition of Grignard reagents to activated 1-acylpyridinium salts to preferentially form 1,2-dihydropyridines, which were then aromatized by a mild oxidation reaction (Scheme 1).

Grignard reagents of **1** were prepared by reaction of magnesium with the appropriate 4′-alkoxy-4-bromobiphenylenes, which were obtained from the reaction of 4′-hydroxy-4-bromobiphenyl with the appropriate bromoalkanes. Reaction of 3-cyanopyridine and phenyl chloroformate produced 3-cyanopyridinium chlorides **2**. Reaction of Grignard **1** with **2** gave the expected 1,2-dihydropyridine adduct **3**. Adducts **3** were then oxidized by treatment with *o*-chloranil to produce the desired 2-(4′-alkoxybiphen-4-yl)-5-cyanopyridine products **4** (*nO-*PPPyCN, *n* = 2–8).

The liquid crystalline compounds **4** were synthesized using a reactant bearing a cyano functional group. Phenyl chloroformate enhances the reactivity of the pyridine ring, facilitates the Grignard’s regioselective nucleophilic attack, and protects the cyano group from the Grignard reagent [22,23].

Our synthetic methodology favors Grignard α-regioselectivity at the pyridine ring. Because of the high polarity difference between the major α and minor γ products, trace amounts of the latter are easily separated using liquid chromatography with a methylene chloride: hexane eluant system in a 3:1 ratio. The yields of this 2-step reaction ranged from 33% to 69% (Table 1).

### 2.2. Thermotropic Studies

**Figure 1:**
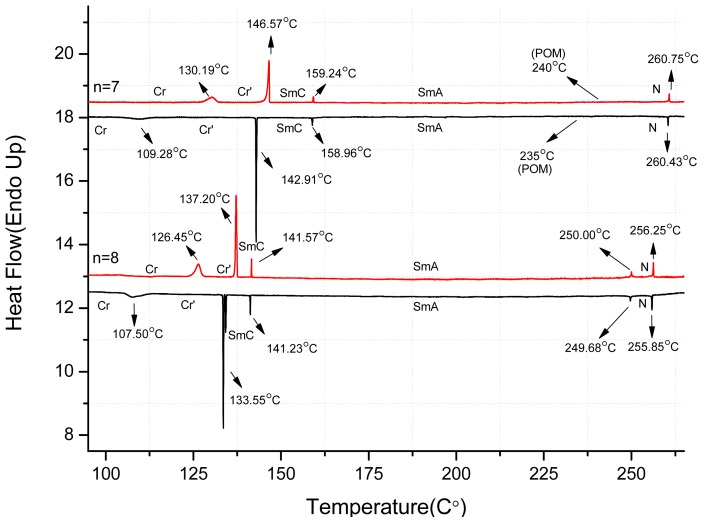
Thermograms of the *nO-*PPPyCN, *n* = 7, 8 obtained using a second DSC scan under a heating (red) and cooling (black) rate of 1 °C min^−1^; Cr, Cr′ = crystal phase; SmC = smectic C phase; SmA = smectic A phase; N = nematic phase; I = isotropic phase.

The phase transition temperatures and associated enthalpy changes of *nO-*PPPyCN were determined using differential scanning calorimetry (DSC). The heating and cooling rates were set to 1 °C min^−1^ because certain phase transition temperatures are sufficiently close that they are not distinguishable at the normal 5 °C min^−1^ scanning rate. Certain crystal-to-crystal transition peaks were observed at temperatures slightly lower than the melting and freezing points for each *nO-*PPPyCN derivative, especially when *n* ≥ 4. The appearance of multiple peaks below the melting point may arise from the motion of the flexible alkoxy chains in *nO-*PPPyCN. Figure 1 shows the DSC thermograms from the second heating and first cooling cycles of 7*O-*PPPyCN and 8*O-*PPPyCN, respectively. The mesophases for these compounds were further identified by observation of their textures using polarized optical microscopy (POM).

All compounds exhibit both enantiotropic nematic and smectic A phases. The longer alkoxy homologues (*n* = 7 and 8) exhibit an additional enantiotropic smectic C phase. The mesomorphic nematic phase appears in a high temperature range of 189–340 °C. The nematic phase ranges of the *n* = 2–4 homologues extend into their decomposition temperatures at approximately 300 °C. Thus, this homologous series of *nO-*PPPyCN liquid crystals has high thermal stability. Table 2 presents a summary of our experimental data.

**Figure 2:**
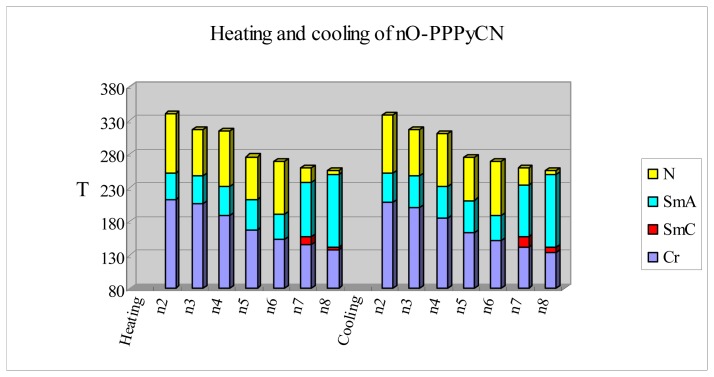
Plot of transition temperatures of heating and cooling cycle for compounds of 2-(4′-alkoxybiphen-4-yl)-5-cyanopyridines (*nO-*PPPyCN, *n* = 2–8).

Figure 2 shows the plot of transition temperatures of the heating and cooling cycle against the number of carbon atoms in the terminal alkoxy chain for the *nO-*PPPyCN homologues. The nematic-to-isotropic transition temperature (*T*_NI_) declines from 340 °C (estimated from POM) to 256.3 °C as the chain lengthens. A pseudo stepwise decrease becomes apparent if the *T*_NI_ values for *n* = 3–8 are grouped into three pairs: *n* = 3 and 4; 5 and 6; and 7 and 8. The values of *T*_NI_ for each pair differ from each other only slightly (317 °C, 314 °C; 276.2 °C, 269.3 °C; 260.8 °C, 256.3 °C, for *n* = 3, 4; 5, 6; and 7, 8, respectively, for heating), similar behaviors were observed under cooling cycles. Typically, the anisotropy of molecular polarizability is greater for alkoxy groups with even members of carbon atoms, and their *T*_NI_ values are greater than their abutting odd membered alkoxy substituents. Thus, the *T*_NI_ temperatures alternate in a zigzag manner as the alkoxy chain length of the series increases [24]. However, in this study the *T*_NI_ value for the shorter odd-numbered homologue is slightly greater than that of its even-numbered partner. We attribute this effect to the existence of relatively strong and narrow (high aspect ratio) anisotropic force fields in the nematic phase of the homologous *nO-*PPPyCN series. Thus, there is a progressive decrease in the anisotropies of molecular polarisability with increasing alkoxy chain length. The observed damping in the stepwise decrease of *T*_NI_ can be accounted for by the statistical increase in the numbers of possible non-extended conformations.

An alternating change in the nematic phase ranges was observed for the *n* = 2–6 homologues. The nematic ranges for the heating cycle are 88.8 °C, 82.3 °C, 79.4 °C, for *n* = 2, 4, 6, and 69.3 °C, 65 °C, for *n* = 3, 5, respectively. Similar behaviors were observed when cooling. The alternating changes seen for the *nO-*PPPyCN nematic phase ranges is a consequence of a strong and narrow (high aspect ratio) anisotropic force field, which is relatively sensitive to the alkoxy chain length in the fluid nematic phase.

On the other hand, the smectic A phase range remains essentially constant with a range covering 36.3–44.3 °C for *n* = 2–6, and increases drastically to 80.8 °C, for *n* = 7, and 108.4 °C for *n* = 8 during heating. Similar behavior occurs during cooling. We assume that competing effects of vibrational motions and attractive forces in the alkoxy chains occur in the smectic A phase. When chain length reaches 7 (heptyl), the alkoxy chain attractive forces substantially outweigh the chain vibrational motions, resulting in the formation of a long-range of smectic A phase. Conversely, short alkoxy chain lengths (*n* = 2–6) tend to disrupt lamella packing.

The enthalpy changes associated with the nematic-smectic A transition, ΔH_N-SmA_, decrease gradually as *n* increases from 3 to 6, and becomes undetectable at *n* = 7. Calculating these values, and scaling by the gas constant, *S*_N-SmA_/*R*, reveals entropy changes of 0.67, 0.36, 0.18, 0.16, and 0 kJ·mol^−1^ for *n* = 3–7, respectively [25,26]. Thus, increasing the value of *n* results in decreases in the magnitude of the entropy change associated with the nematic to smectic A transition. Apparently, in the nematic phase the shorter the alkoxy chain, the greater its mobility (except for *n* = 2, where it is too short to exhibit a high mobility change between the nematic and smectic A phases). The mobility of the alkoxy chain becomes more restricted as the compound is cooled to smectic A phase. The attractive forces of the alkoxy chain increase with increasing chain length, which results in a decreasing trend for the nematic to smectic A transition entropy change. Zero enthalpy and entropy change for the transition is achieved at *n* = 7, indicating that little change in chain conformations occurs between the nematic and smectic A phases. Nevertheless, the nematic to smectic A phase transition of 7*O-*PPPyCN was observed using POM.

**Figure 3:**
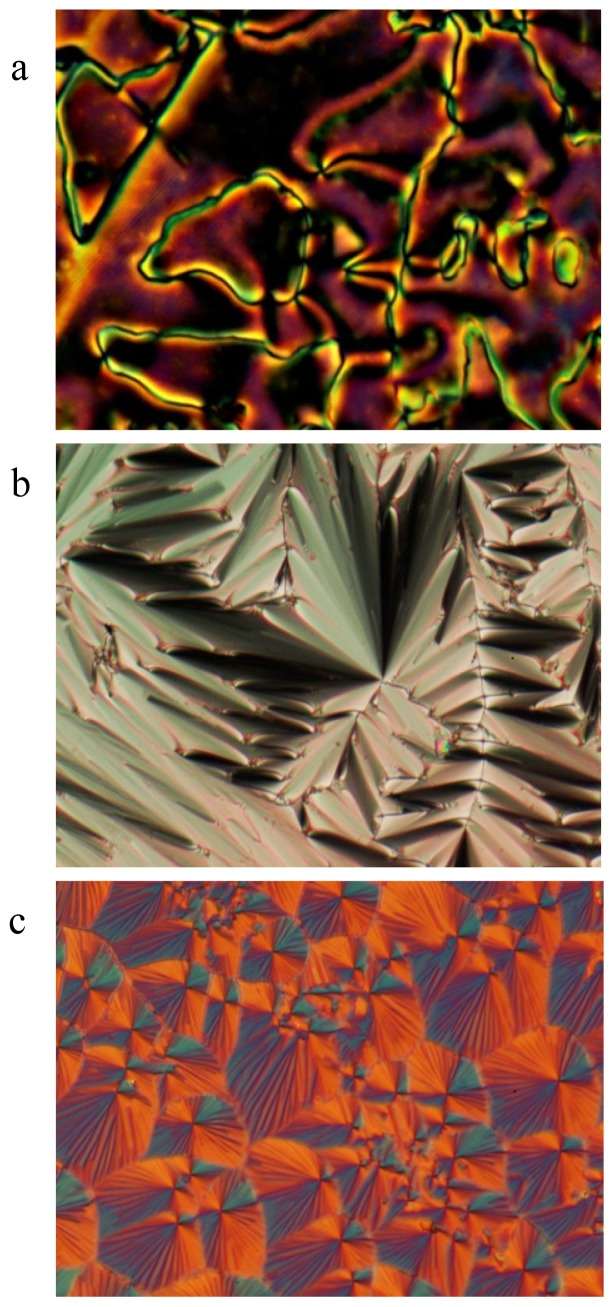
Polarized optical micrographs of 7*O-*PPPyCN arise from isotropic phase on cooling to (**a**) 246 °C, nematic Schlieren texture, ×400; (**b**) 162 °C, smectic A texture, ×200; (**c**) 148 °C, smectic C texture, ×200.

Short temperature ranges of 12.6 °C and 4.4 °C of enantiotropic smectic C phase appear on heating when *n* = 7 and 8, respectively. The appearance of smectic C phase for *n* = 7 and 8 provides further evidence for the participation of the alkoxy chain in the formation of lamella packing. The enthalpy changes associated with smectic C to smectic A transition are in the range of 0.39–0.72 kJ·mol^−1^, which are relatively high enthalpies for this type of transition [12]. Micrographs of the nematic Schlieren texture, the focal conic texture of the smectic A phase, and the broken focal conic texture of the smectic C phase of 7*O-*PPPyCN arising from the isotropic phase on cooling are shown respectively in Figure 3a–c.

The 4-alkoxy-4′-cyano-*p*-terphenyl (*xO-*PPPCN, *x* = 2–7) benzene analogues were synthesized and investigated twelve years ago [27]. All of the *xO-*PPPCN homologues exhibit both nematic phase and smectic phases. The 2*O-*PPPCN, 3*O-*PPPCN, and 4*O-*PPPCN homologues each show one smectic phase (SmB for *x* = 2, 3 and SmX for *x* = 4) and one nematic. The 5*O-*PPPCN, 6*O-*PPPCN, and 7*O-*PPPCN homologues, each exhibit two smectic phases (SmF, SmB) and one nematic phase. Polymesomorphism increases with *x* in these linear mesogens.

**Figure 4:**
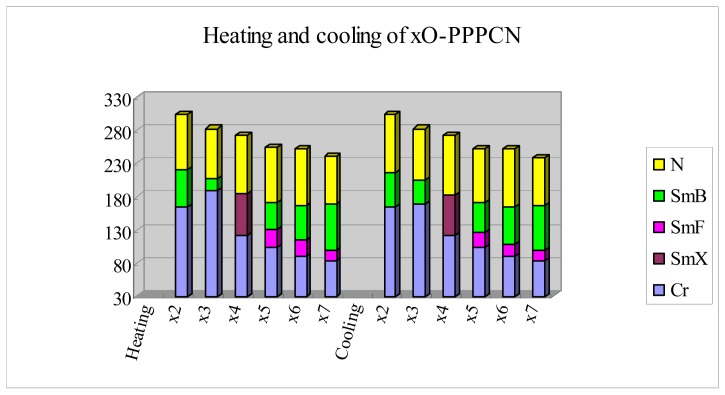
Plot of transition temperatures of heating and cooling cycle for compounds of 4-alkoxy-4′-cyano-*p*-terphenyls (*xO-*PPPCN, *x* = 2–7).

Homologues of *xO-*PPPCN have both lower melting points (83–190 °C *vs.* 137–212 °C) and lower nematic-to-isotropic transition temperatures (240–304 °C *vs.* 256–340 °C) compared with the *nO-*PPPyCN compounds. Apparently, the strong intermolecular dipolar attractive force of the cyanopyridyl terminus in *nO-*PPPyCN increases both the melting temperature and the nematic-to-isotropic transition temperature relative to those seen in the *xO-*PPPCNs. However, highly ordered SmF and SmB phases were observed in the *xO-*PPPCN series (Figure 4) and less ordered SmC and SmA phases appeared in the *nO-*PPPyCN series. Although pyridine-containing liquid crystalline molecules typically increase molecular lateral attractive forces and favor smectic behavior [28], our results show that replacement of a phenyl ring with a pyridyl ring does not result in the formation of a highly ordered smectic phase. Thus, we assume that the alignment of the terphenyl moiety along the molecular long axis in *xO-*PPPCN plays a key role in generation of the highly ordered SmF and SmB phases. By contrast, the low order smectic phases, SmC and SmA, emerge because the combination of the non-equilateral hexagonal symmetry of the pyridyl moiety and the asymmetrical alignment of the pyridyl dipole with the *nO-*PPPyCN long molecular axis result in orienting the terminal cyano group slightly away from the long molecular axis.

Under the DSC heating cycle, the lengths of nematic phase for *xO-*PPPCN, with *x* = 2, 3, 4, 5, 6 are 83 °C, 75 °C, 87 °C, 81 °C, and 84 °C, respectively; these lengths are approximately equivalent to those 88.8 °C, 69.3 °C, 82.3 °C, 65.0 °C, and 79.4 °C, for *nO-*PPPyCN, with *n* = 2, 3, 4, 5, 6, respectively. We attribute these similarities to the equivalent aspect ratios of the three-aryl-ring mesogenic cores that are common to both sets of compounds. It is noteworthy also that the alternation in T_NI_ does not occur in the *xO-*PPPCN series. Thus, we observed that the T_NI_ values for the shorter odd-numbered alkoxy chains are slightly greater than those of the adjacent longer even-numbered homologues.

**Figure 5:**
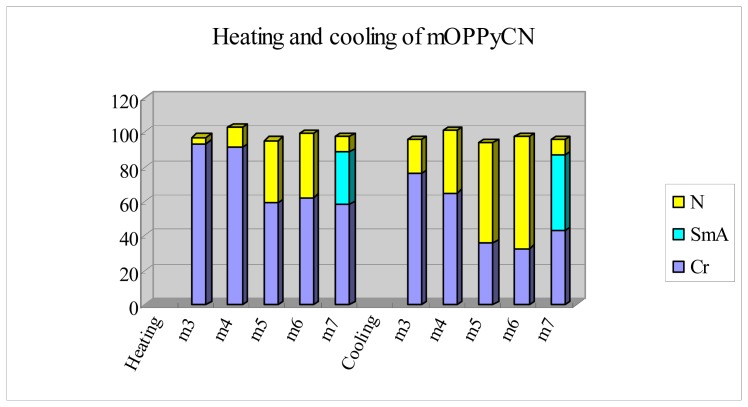
Plot of transition temperatures of heating and cooling cycle for compounds of 2-(4-alkyloxyphenyl)-5-cyanopyridines (*mO-*PPyCN, *m* = 3–7).

Early experimental results on the 2-(4-alkoxyphenyl)-5-cyanopyridine (*mO-*PPyCN, *m* = 3–7) homologues suggest that the presence of a pyridine moiety brings about the early appearance of both the smectic phase for *m* = 7 and also the nematic phase for *m* = 3 and 4 [19] (Figure 5). Compared with the series of *mO-*PPyCN homologues, the presence of an extra phenyl moiety in the *nO-*PPPyCN series increases both melting temperature and the nematic-to*-*isotropic transition temperature. The enhancement is most pronounced for the nematic-to-isotropic transition (95–103 °C for *mO-*PPyCNs *vs.* 256.3–340 °C for *nO-*PPPyCN), and is less marked for the melting transition (58–94 °C for *mO-*PPyCN *vs.* 137.2–212.4 °C in *nO-*PPPyCN). We attribute this difference in enhancement to the high aspect ratio of the three-aryl-ring mesogenic core, which stabilizes the nematic phase. However, an early, persistent appearance of the smectic A phase was also observed in the *nO-*PPPyCN homologues. Mesomorphic states become more favorable with increasing aspect ratio of the mesogenic core, thus, greater anisotropic polarizability arises as the length of the (teraryl) mesogen is increased.

**Figure 6:**
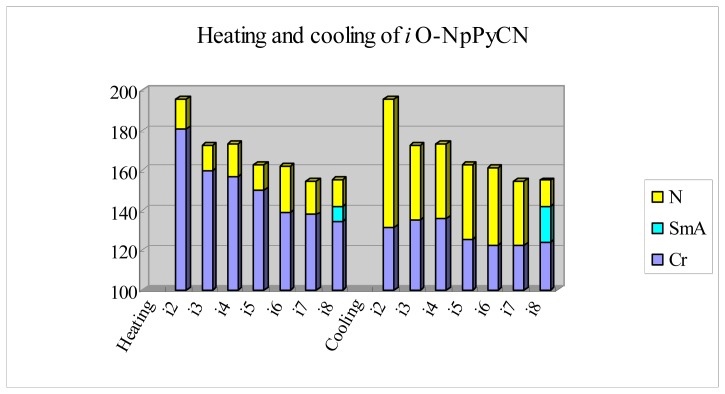
Plot of transition temperatures of heating and cooling cycle for compounds 2-(6-alkoxynaphthalen-2-yl)-5-cyanopyridines (*iO-*NpPyCN, *i* = 2–8).

The mesomorphic properties of 2-(6-alkoxynaphthalen-2-yl)-5-cyanopyridines, (*iO-*NpPyCN, *i* = 2–8) (Figure 6) can also be compared with those of *nO-*PPPyCNs [29]. The non-symmetrical 2,6-naphthalene core in *iO-*NpPyCN reduces not only crystalline packing, resulting in a low melting point, but also prohibits its smectic layered-structure. Thus, a nematic phase with a broad mesomorphic range was observed in the *iO-*NpPyCN series. This finding provides solid evidence that polymesomorphism exhibited by the *nO-*PPPyCN homologues originates from their linear mesogenic core. Thus, pyridine-containing LC compounds tend to be polymesomorphic only if the molecule has a linear mesogenic structure. Furthermore, enthalpies for the nematic-to-isotropic phase transition in the *iO-*NpPyCN series are of the order 0.3 kJ·mol^−1^. The small transitional enthalpy changes seen for the *nO-*NpPyCN series imply loose intermolecular packing in the nematic phase. By contrast, the enthalpy changes associated with nematic-to-isotropic transitions seen for the *nO-*PPPyCN series are in the range of 0.55–1.35 kJ·mol^−1^, reflecting the presence of a relatively strong anisotropic force field in the *nO-*PPPyCN nematic phase. Thus, we can conclude that the fluid nematic phase is favored by linear molecular structures with one kink (naphthalene) in the mesogenic core.

## 3. Experimental Section

### 3.1. General

The chemical structures of the compounds were analyzed by ^1^H and ^13^C-NMR spectra using a Bruker AC 300 spectrometer (Bruker Corporation, Billerica, MA, USA). Infrared (IR) spectra were recorded on a Perkin-Elmer 1600 Series spectrometer (Perkin-Elmer, Norwalk, CT, USA). The purity of the compounds was checked by thin-layer chromatography and further confirmed by elemental analysis.

Mesophases were chiefly identified by the microscopic texture of samples sandwiched between two glass plates under a polarizing optical microscope (POM; Olympus BH-2, Two Corporate Center Drive, Melville, NY, USA) equipped with a Mettler FP90/FP82HT hot stage (Mettler, Columbus, OH, USA). Phase transition temperatures and their corresponding transition enthalpies were determined by differential scanning calorimetry (DSC), using a Perkin-Elmer DSC 7 calorimeter at a scanning rate of 1 °C·min^−1^. The program consists of first heating followed by repeated cooling and heating cycles. Data from the repeated cycles were found to be identical to those of the first cooling and second heating. Therefore, only the data from the first cooling and second heating cycles are reported.

### 3.2. Synthesis

The starting materials, 4-bromo-4′-hydroxybiphenyl, and 3-cyanopyridine were purchased from Aldrich Chemical Co. (Saint Louis, MO, USA) and used as-received. Phenyl chloroformate and *n*-bromoalkanes were distilled under an inert nitrogen atmosphere immediately before use. Anhydrous organic solvents, toluene and tetrahydrofuran (THF), were first heated at reflux over sodium and then distilled under nitrogen before use. Column chromatography was carried out with silica gel (MN Kieselgel 60, 70–230 mesh, Duren, Germany). The purity of the compounds was checked by thin-layer chromatography and further confirmed by elemental analysis. The synthesis of 2-(4′-alkoxybiphen-4-yl)-5-cyanopyridines was carried out according to the synthetic methods outlined in Scheme 1.

#### 3.2.1. Representative procedure for the homologues of 2-(4′-alkoxybiphen-4-yl)-5-cyanopyridines (*nO-*PPPyCN, *n* = 2–8)

The entire synthetic procedures were completed in a short two-step process with overall yields in the range of 33%–69% (Table 1). For 2*O-*PPPyCN in **3**, to a solution of 4-bromo-4′-ethoxybiphenyl (10 mmol) in THF (20 mL), freshly dried magnesium granules (11 mmol) were added under an inert nitrogen atmosphere over about half an hour. The Grignard solution **1** was then slowly added by a syringe into a preformed solution of 3-cyanopyridinium chloride **2**, which was prepared from phenyl chloroformate (10 mmol) and 3-cyanopyridine (10 mmol) in dry THF (20 mL) at −20 °C for 0.5 h. The resulting solution was heated slowly to room temperature and stirred for another 8 h. After the solvent THF was evaporated, the residue was extracted with Et_2_O. The organic layer was further washed once with 20% NH_4_Cl solution and twice with distilled water and brine and dried with magnesium sulfate. For 2*O-*PPPyCN in **4**, to a solution of dry toluene (20 mL) and compound **3** (10 mmol), about 1.5 eq. *o*-chloranil was added. The reaction mixture was heated to reflux for about 3 h under inert nitrogen atmosphere and then quenched by adding 1N NaOH (25 mL) and Et_2_O (25 mL) and filtered through Celite (Duren, Germany). Normal aqueous work-up and isolation with column chromatography (methylene chloride:hexane = 3:1) afford an overall two-step reaction with good yield of 2-(4′-ethoxybiphen-4-yl)-5-cyanopyridine **4** (69%). The crude products of **4** were further purified by recrystallization from a mixed solvent of methylene chloride and ethyl acetate. The other *nO-*PPPyCN homologues were synthesized essentially by the same procedure as described above for the *n* = 2 homologue. All compounds gave satisfactory ^1^H-NMR, ^13^C-NMR, IR and elemental analysis results as discussed in the following section.

##### 3.2.1.1. 2-(4-Ethoxybiphen-4′-yl)-5-cyanopyridine (2*O-*PPPyCN)

^1^H-NMR (CDCl_3_): δ 8.95 (dd, 1H, *J*_1_ = 2.1 Hz, *J*_2_ = 0.9 Hz, pyridine), 8.11 (d, 2H, *J* = 8.4 Hz, center-benzene), 8.00 (dd, 1H, *J*_1_ = 8.4 Hz, *J*_2_ = 2.1 Hz, pyridine), 7.88 (dd, 1H, *J*_1_ = 8.4 Hz, *J*_2_ = 0.9 Hz, pyridine), 7.71 (d, 2H, *J* = 8.4 Hz, center-benzene), 7.59 (d, 2H, *J* = 8.7 Hz, outer-benzene), 7.00 (d, 2H, *J* = 8.7 Hz, outer-benzene), 4.10 (q, 2H, *J* = 7.0 Hz, –CH_2_), 1.46 (t, 3H, *J* = 6.9 Hz, –CH_3_). ^13^C-NMR (CDCl_3_): ppm 160.2, 159.2, 152.6, 143.1, 139.9, 135.5, 132.3, 128.2, 127.8, 127.2, 119.7, 117.2, 115.0, 107.6, 63.6, 14.9. IR (KBr): cm^−1^ 3041 (aromatic C–H stretch), 2984 (aliphatic C–H asymmetric stretch), 2231 (nitrile C≡N stretch), 1584 (ring stretch), 1468 (ring stretch), 1249 (asymmetric C–O–C stretch), 1041 (symmetric C–O–C stretch), 821 (out-of-plane C–H bend), 763 (out-of-plane C–H bend). Anal. calcd for C_20_H_16_N_2_O: C, 79.98; H, 5.37; N, 9.33. Found: C, 79.76; H, 5.39; N, 9.23.

##### 3.2.1.2. 2-(4-propoxybiphen-4′-yl)-5-cyanopyridine (3*O-*PPPyCN)

^1^H-NMR (CDCl_3_): δ 8.95 (d, 1H, *J* = 1.2 Hz, pyridine), 8.10 (d, 2H, *J* = 8.4 Hz, center-benzene), 8.00 (dd, 1H, *J*_1_ = 8.4 Hz, *J*_2_ = 2.1 Hz, pyridine), 7.87 (dd, 1H, *J*_1_ = 8.1 Hz, *J*_2_ = 0.6 Hz, pyridine), 7.70 (d, 2H, *J* = 8.4 Hz, center-benzene), 7.59 (d, 2H, *J* = 8.7 Hz, outer-benzene), 7.00 (d, 2H, *J* = 8.7 Hz, outer-benzene), 3.98 (t, 2H, *J* = 6.6 Hz, –CH_2_), 1.85 (sext, 2H, *J* = 7.1 Hz, –CH_2_), 1.07 (t, 3H, *J* = 7.5 Hz, –CH_3_). ^13^C-NMR (CDCl_3_): ppm 160.2, 159.4, 152.6, 143.2, 139.9, 135.5, 132.3, 128.2, 127.9, 127.2, 119.8, 117.2, 115.0, 107.7, 69.7, 22.7, 10.6. IR (KBr): cm^−1^ 3041 (aromatic C–H stretch), 2973 (aliphatic C-H asymmetric stretch), 2233 (nitrile C≡N stretch), 1586 (ring stretch), 1469 (ring stretch), 1252 (asymmetric C–O–C stretch), 1065 (symmetric C–O–C stretch), 815 (out-of-plane C–H bend), 763 (out-of-plane C–H bend). Anal. calcd for C_21_H_18_N_2_O: C, 80.23; H, 5.77; N, 8.91. Found: C, 80.19; H, 5.80; N, 8.86.

##### 3.2.1.3. 2-(4-butoxybiphen-4′-yl)-5-cyanopyridine (4*O-*PPPyCN)

^1^H-NMR (CDCl_3_): δ 8.95 (d, 1H, *J* = 1.8 Hz, pyridine), 8.11 (d, 2H, *J* = 8.4 Hz, center-benzene), 8.01 (dd, 1H, *J*_1_ = 8.1 Hz, *J*_2_ = 2.1 Hz, pyridine), 7.88 (d, 1H, *J* = 8.4 Hz, pyridine), 7.71 (d, 2H, *J* = 8.4 Hz, center-benzene), 7.59 (d, 2H, *J* = 8.7 Hz, outer-benzene), 7.00 (d, 2H, *J* = 8.7 Hz, outer-benzene), 4.02 (t, 2H, *J* = 6.5 Hz, –CH_2_), 1.81 (quin, 2H, *J* = 7.1 Hz, –CH_2_), 1.52 (sext, 2H, *J* = 7.0 Hz, –CH_2_), 1.00 (t, 3H, *J* = 7.4 Hz, –CH_3_). ^13^C-NMR (CDCl_3_): ppm 160.2, 159.4, 152.6, 143.2, 139.9, 135.5, 132.3, 128.2, 127.8, 127.2, 119.8, 117.2, 115.0, 107.7, 67.9, 31.4, 19.3, 13.9. IR (KBr): cm^−1^ 3040 (aromatic C–H stretch), 2957 (aliphatic C–H asymmetric stretch), 2873 (aliphatic C–H asymmetric stretch), 2235 (nitrile C≡N stretch), 1589 (ring stretch), 1471 (ring stretch), 1253 (asymmetric C–O–C stretch), 1023 (symmetric C–O–C stretch), 817 (out-of-plane C–H bend), 764 (out-of-plane C–H bend). Anal. calcd for C_22_H_20_N_2_O: C, 80.46; H, 6.14; N, 8.53. Found: C, 80.40; H, 6.14; N, 8.50.

##### 3.2.1.4. 2-(4-pentoxybiphen-4′-yl)-5-cyanopyridine (5*O-*PPPyCN)

^1^H-NMR (CDCl_3_): δ 8.95 (d, 1H, *J* = 1.2 Hz, pyridine), 8.10 (d, 2H, *J* = 8.4 Hz, center-benzene), 8.00 (dd, 1H, *J*_1_ = 8.4 Hz, *J*_2_ = 1.8 Hz, pyridine), 7.87 (d, 1H, *J* = 8.4 Hz, pyridine), 7.70 (d, 2H, *J* = 8.4 Hz, center-benzene), 7.59 (d, 2H, *J* = 8.7 Hz, outer-benzene), 7.00 (d, 2H, *J* = 8.7 Hz, outer-benzene), 4.01 (t, 2H, *J* = 6.6 Hz, –CH_2_), 1.83 (quin, 2H, *J* = 6.9 Hz, –CH_2_), 1.35–1.52 (m, 4H, –CH_2_), 0.95 (t, 3H, *J* = 7.1 Hz, –CH_3_). ^13^C-NMR (CDCl_3_): ppm 160.2, 159.4, 152.6, 143.2, 139.9, 135.5, 132.3, 128.2, 127.8, 127.2, 119.8, 117.2, 115.0, 107.7, 68.2, 29.0, 28.3, 22.6, 14.1. IR (KBr): cm^−1^ 3040 (aromatic C–H stretch), 2931 (aliphatic C–H asymmetric stretch), 2861 (aliphatic C–H asymmetric stretch), 2235 (nitrile C≡N stretch), 1591 (ring stretch), 1469 (ring stretch), 1251 (asymmetric C–O–C stretch), 1031 (symmetric C–O–C stretch), 812 (out-of-plane C–H bend), 765 (out-of-plane C–H bend). Anal. calcd for C_23_H_22_N_2_O: C, 80.67; H, 6.48; N, 8.18. Found: C, 80.58; H, 6.51; N, 8.08.

##### 3.2.1.5. 2-(4-hexoxybiphen-4′-yl)-5-cyanopyridine (6*O-*PPPyCN)

^1^H-NMR (CDCl_3_): δ 8.95 (d, 1H, *J* = 1.8 Hz, pyridine), 8.11 (d, 2H, *J* = 8.4 Hz, center-benzene), 8.00 (dd, 1H, *J*_1_ = 8.4 Hz, *J*_2_ = 2.1 Hz, pyridine), 7.87 (d, 1H, *J* = 8.4 Hz, pyridine), 7.71 (d, 2H, *J* = 8.4 Hz, center-benzene), 7.59 (d, 2H, *J* = 8.7 Hz, outer-benzene), 7.00 (d, 2H, *J* = 8.7 Hz, outer-benzene), 4.01 (t, 2H, *J* = 6.6 Hz, –CH_2_), 1.82 (quin, 2H, *J* = 7.1 Hz, –CH_2_), 1.33–1.51 (m, 6H, –CH_2_), 0.92 (t, 3H, *J* = 7.1 Hz, –CH_3_). ^13^C-NMR (CDCl_3_): ppm 160.2, 159.4, 152.6, 143.2, 139.9, 135.5, 132.3, 128.2, 127.9, 127.2, 119.8, 117.2, 115.0, 107.7, 68.2, 31.7, 29.3, 25.8, 22.7, 14.1. IR (KBr): cm^−1^ 3040 (aromatic C–H stretch), 2956 (aliphatic C–H asymmetric stretch), 2931 (aliphatic C–H asymmetric stretch), 2859 (aliphatic C–H asymmetric stretch), 2235 (nitrile C≡N stretch), 1591 (ring stretch), 1470 (ring stretch), 1251 (asymmetric C–O–C stretch), 1034 (symmetric C–O–C stretch), 815 (out-of-plane C–H bend), 765 (out-of-plane C–H bend). Anal. calcd for C_24_H_24_N_2_O: C, 80.87; H, 6.79; N, 7.86. Found: C, 80.90; H, 6.85; N, 7.71.

##### 3.2.1.6. 2-(4-heptoxybiphen-4′-yl)-5-cyanopyridine (7*O-*PPPyCN)

^1^H-NMR (CDCl_3_): δ 8.95 (dd, 1H, *J*_1_ = 2.1 Hz, *J*_2_ = 0.6 Hz, pyridine), 8.10 (d, 2H, *J* = 8.7 Hz, center-benzene), 8.00 (dd, 1H, *J*_1_ = 8.4 Hz, *J*_2_ = 2.1 Hz, pyridine), 7.87 (dd, 1H, *J*_1_ = 8.4 Hz, *J*_2_ = 0.6 Hz, pyridine), 7.70 (d, 2H, *J* = 8.7 Hz, center-benzene), 7.59 (d, 2H, *J* = 8.7 Hz, outer-benzene), 7.00 (d, 2H, *J*_1_ = 8.7 Hz, outer-benzene), 4.01 (t, 2H, *J* = 6.6 Hz, –CH_2_), 1.82 (quin, 2H, *J* = 7.0 Hz, –CH_2_), 1.33–1.51 (m, 8H, –CH_2_), 0.90 (t, 3H, *J* = 6.6 Hz, –CH_3_). ^13^C-NMR (CDCl_3_): ppm 160.2, 159.4, 152.6, 143.2, 139.9, 135.5, 132.3, 128.2, 127.9, 127.2, 119.8, 117.2, 115.0, 107.7, 68.2, 31.9, 29.4, 29.2, 26.1, 22.7, 14.2. IR (KBr): cm^−1^ 3040 (aromatic C–H stretch), 2954 (aliphatic C–H asymmetric stretch), 2930 (aliphatic C–H asymmetric stretch), 2859 (aliphatic C–H asymmetric stretch), 2236 (nitrile C≡N stretch), 1592 (ring stretch), 1471 (ring stretch), 1253 (asymmetric C–O–C stretch), 1043 (symmetric C–O–C stretch), 812 (out-of-plane C–H bend), 765 (out-of-plane C–H bend). Anal. calcd for C_25_H_26_N_2_O: C, 81.05; H, 7.07; N, 7.56. Found: C, 81.02; H, 7.11; N, 7.57.

##### 3.2.1.7. 2-(4-octoxybiphen-4′-yl)-5-cyanopyridine (8*O-*PPPyCN)

^1^H-NMR (CDCl_3_): δ 8.95 (s, 1H, pyridine), 8.11 (d, 2H, *J* = 8.4 Hz, center-benzene), 8.00 (dd, 1H, *J*_1_ = 8.4 Hz, *J*_2_ = 2.1 Hz, pyridine), 7.88 (d, 1H, *J* = 8.4 Hz, pyridine), 7.71 (d, 2H, *J* = 8.4 Hz, center-benzene), 7.59 (d, 2H, *J* = 8.7 Hz, outer-benzene), 7.00 (d, 2H, *J* = 9.0 Hz, outer-benzene), 4.01 (t, 2H, *J* = 6.6 Hz, –CH_2_), 1.82 (quin, 2H, *J* = 7.0 Hz, –CH_2_), 1.30–1.51 (m, 10H, –CH_2_), 0.90 (t, 3H, *J* = 6.8 Hz, –CH_3_). ^13^C-NMR (CDCl_3_): ppm 160.2, 159.4, 152.6, 143.2, 139.9, 135.5, 132.3, 128.2, 127.8, 127.2, 119.8, 117.2, 115.0, 107.7, 68.2, 31.9, 29.4, 29.3, 26.1, 22.7, 14.2. IR (KBr): cm^−1^ 3041 (aromatic C–H stretch), 2918 (aliphatic C–H asymmetric stretch), 2853 (aliphatic C–H asymmetric stretch), 2248 (nitrile C≡N stretch), 1592 (ring stretch), 1471 (ring stretch), 1253 (asymmetric C–O–C stretch), 1047 (symmetric C–O–C stretch), 814 (out-of-plane C–H bend), 765 (out-of-plane C–H bend). Anal. calcd for C_26_H_28_N_2_O: C, 81.21; H, 7.34; N, 7.29. Found: C, 81.12; H, 7.26; N, 7.19.

## 4. Conclusions

In this study, we reported the convenient, short 2-step synthesis of a novel series of liquid crystalline compounds, *nO-*PPPyCN, where *n* = 2–8, with fair to good 2-step overall yields of 33%–69%. The short synthesis and good yields provide ready access to these compounds and will facilitate the further study of their thermotropic properties.

The entire range of *nO-*PPPyCN homologues, with *n* = 2–8, exhibits both enantiotropic nematic and smectic A phases, along with an additional enantiotropic smectic C phase for the longer alkoxy homologues with *n* = 7 and 8. Polymesomorphism was observed in the liquid crystals with linear structures. We compared the mesomorphic behaviors of the target *nO-*PPPyCN molecules with those of *xO-*PPPCN, *mO-*PPyCN and *iO-*NpPyCN. We observed that smectic phases with low order were favored by *nO-*PPPyCN because its pyridyl moiety aligns asymmetrically along the molecular long axis, disrupting mesomorphic lamellar packing. The high-aspect-ratio of the linear three-aryl-ring mesogenic core in *nO-*PPPyCN contributes substantially to enhancement of T_NI_ and melting temperature. The fluid nematic phase is favored by a linear molecular structure with one kink provided by naphthalene (as in *iO-*NpPyCN) in its mesogenic core.

## Figures and Tables

**Table 1:** Yields of 2-(4′-alkoxybiphen-4-yl)-5-cyanopyridines (*nO-*PPPyCN, *n* = 2–8).

Entry (*n*)	Alkyl group	Yield a(%)
2	Ethyl	69
3	Propyl	47
4	Butyl	33
5	Pentyl	39
6	Hexyl	38
7	Heptyl	36
8	Octyl	34

a Isolated yields are quoted after recrystallization.

**Table 2:** Phase transition temperatures (°C) and corresponding transition enthalpies (kJ·mol^−1^), in parentheses, for homologous series of *nO-*PPPyCN, *n* = 2–8.

Compound *nO-*PPPyCN (*n*)	Phase transition temperatures (°C) and theircorresponding transition enthalpies (kJ·mol^−1^)	

	Heating	Cooling
2	Cr 212.4(8.72) SmA 251.2(2.14) N 340 a I	I 338 a N 250.8(2.05) SmA 208.8(8.25) Cr
3	Cr 206.3(12.02) SmA 247.7(2.93) N 317 a I	I 316 a N 247.1(2.94) SmA 201.4(11.36) Cr
4	Cr 188.3(~12.19 b) SmA 231.7(1.53) N 314 a I	I 310 a N 231.3(1.23) SmA 184.1(~12.98^b^) Cr
5	Cr′ 157.9(7.33) Cr 167.4(7.51) SmA 211.7(0.73) N 276.2(0.58) I	I 275.7(0.64) N 211.3(0.78) SmA 163.8(~7.56 b) Cr 150.6(7.15) Cr′
6	Cr′ 145.3(5.10) Cr 153.6(8.82) SmA 189.9(0.63) N 269.3(0.92) I	I 268.8(0.77) N 189.6(0.16) SmA 150.8(~8.58 b) Cr 133.6(4.17) Cr′
7	Cr′ 130.2(3.49) Cr 146.6(7.18) SmC 159.2(0.39) SmA 240 c N 260.8(0.55) I	I 260.4(0.58) N 235 c SmA 159.0(0.58) SmC 142.9(6.93) Cr 109.3(3.01) Cr′
8	Cr′ 126.5(6.21) Cr 137.2(9.87) SmC 141.6(0.72) SmA 250.0(0.81) N 256.3(1.35) I	I 255.9(1.09) N 249.7(0.56) SmA 141.2(0.66) SmC 133.6(~9.48 b) Cr 107.5(4.68) Cr′

a The temperature was obtained using polarized optical microscopy (POM) because of the sample’s decomposition;

b Several peaks cluster near this temperature, the enthalpies are added altogether accordingly since there is no significant difference observed under POM;

c The temperatures were obtained from POM by a linear intrapolation from the comparison of data between DSC and POM; Scan rate: 1 °C min^−1^ for all samples;

Cr, Cr′ = crystal phases, SmC = smectic C phase, SmA = smectic A phase, N = nematic phase, I = isotropic phase.
